# Preparatory routines for emotional regulation in performance enhancement

**DOI:** 10.3389/fpsyg.2022.948512

**Published:** 2022-07-29

**Authors:** Iris Orbach, Boris Blumenstein

**Affiliations:** ^1^College of Management Academic Studies, Rishon LeZion, Israel; ^2^Ribstein Research Center, Wingate Institute, Netanya, Israel

**Keywords:** periodization, LMA, biofeedback training, preparatory routine, self-paced

## Abstract

Preparatory routines (PR) are a necessary tool for achieving optimal emotional states and effective performance, especially in self-paced motor skills. Numerous studies in the area of applied sport psychology have found a positive effect of PR on blocking out distractions and regulating performance thoughts, actions, and emotions. PR contain behavioral and mental components that can be applied in different time periods before and after the event itself: pre-competitive activity routines (PCA-R), pre-performance routines (PP-R), and post-performance activity routines (PPA-R). The aim of this manuscript is to present an effective way to practice psychological techniques and their combinations as part of the PR. The periodization principle and the Learning-Modification-Application (LMA) model with biofeedback training can provide a conceptual framework and means of application for performance enhancement. It will allow the transfer of psychological skills from lab to field and the integration of PR into the athlete’s preparations for peak performance.

## Introduction

Peak performance in sport is usually associated with personal best results. A major condition for achieving peak performance in elite sports is for the athlete to learn how to attain and preserve an optimal state under competition stress distractions. An optimal state does not happen by itself; it requires a long and involved training process aimed at learning, developing, and practicing psychological skills and strategies (Swann et al., 2012, 2017).

In modern sport, many stress distractions interfere with emotions, and therefore have a dominant effect on performance. Especially during the days immediately before competition, the physical load decreases while the emotional/mental tension increases (Blumenstein and Weinstein, 2010; Bompa et al., 2019). Routine is a strategy to regulate emotion, bringing together the application of mental skills and strategies to enhance an athlete’s performance while integrating elements of their repertoire during various competitions (Lidor, 2007; Jackson, 2014). The aim of this article is to demonstrate a conceptual framework, the 5-stage PST model, for emotional regulation using preparatory routines. A case study based on the 5-stage PST model of practicing preparatory routines in a self-paced motor task is presented below.

## Preparatory routines

Preparatory routines (PR) are patterns of physical and mental actions that athletes use before performing. More specifically, PR are learned and practiced in order to direct the athlete’s attention, help regulate psychological and physiological responses to stress, and allow motor processes to run with minimal conscious interference (Jackson, 2014; Bompa et al., 2019). Research has suggested that PR can focus an athlete’s attention, helping them concentrate on the relevant aspects of the task and block out distractions (Cotterill, 2010; Rupprecht et al., 2021). In addition, PR help athletes regulate their emotions, thoughts, and behaviors so that they can minimize distractions and focus on performing motor tasks optimally (Jackson, 2014). Finally, these routines can help trigger automatic performance, which can facilitate a task’s accuracy, especially self-paced tasks (Lidor, 2007; Cotterill, 2010). During this period the athlete puts together relevant psychological skills/strategies (i.e., relaxation, imagery, concentration, positive self-talk) with physical activities, which allows the athlete to achieve an optimal level of arousal/activation (i.e., flow), and, finally, performance best. PR contain behavioral and mental components that can be applied during different time periods before and after the event itself: *pre-competitive activity routine* (PCA-R), *pre-performance routine* (PP-R), and *post-performance activity routine* (PPA-R).

### Pre-competitive activity routine

The day before a competition, the athlete performs a PCA-R, which includes special procedures concerning actions, thoughts, and rituals. The PCA-R helps athletes achieve an optimal feeling of mental readiness, regulates emotions, and increases self-confidence. It is based on the athlete’s retrospective performance experiences, from which they select various elements of precompetitive activity, actions, thoughts, and psychological strategies that correlate with successful results from the past. Examples might include relaxation, self-talk, imagery, habitual clothes, sitting in a special place on the bus, eating specific food, length and content of warm-up, and various routines and rituals to be performed the day before competition. In team sports, the athlete correlates their personal routine with the structure and schedule of the team’s preparation, for example, team meetings, small group meetings (e.g., defense, offense), talking with the coach, and team warm-up (Rupprecht et al., 2021).

### Pre-performance routine

A short time before the actual performance in competition, the athlete can use a PP-R to create an optimal body-mind state for peak performance. PP-R is a significant part of athletic performance, during which the athlete utilizes their plan of behavior, thoughts, and feelings to prepare for the performance. In general, PP-R is a ritual scenario of what an athlete/team does, thinks, and feels to get into the “zone” prior to the actual performance. Many examples of PP-R in various sports are described in the psychological literature (e.g., Lidor, 2007; Cotterill, 2010; Gropel and Beckmann, 2017). For example, PP-R for a free throw in basketball may include holding the ball, self-talking, dribbling, or focusing attention on the basket before the actual throw. The athlete demonstrates their PP-R automatically, quickly, and precisely. All psychological techniques applied in this time period must be short (e.g., relaxation 5–10 s), punctual, and combined at exactly the right time, with full concentration and high confidence. Finally, PP-R has individual peculiarities linked with the athlete’s experience and with the demands of the competition.

### Post-performance activity routine

Lastly, post-performance activity routines (PPA-R) take place following the performance. They include analysis of the performance and actions to prepare the athlete for the next attempt, which might start a few minutes or a few hours after the previous one (e.g., fights in combat sport, jumps in athletics). During this period, the athlete’s first self-estimates as to what has just happened emphasize positive elements for upcoming performance, and in some cases reach a conclusion as to what needs to be improved in the next attempt/fight/throw (Lidor, 2007).

## Psychological skills training

Preparatory routines are specific to each athlete and sport discipline, combine a number of psychological skills, and place the mind in a condition that facilitates its readiness to allow the body to perform (Henschen, 2005). Generally, the psychological strategies the athlete uses in practice should be systematically performed and modified according to competition demands. This can be achieved by integration of psychological skills training (PST) with athlete’s preparation, using routines as an important means to facilitate the process (Bompa et al., 2019). To achieve this goal, Blumenstein and Orbach (2014, 2018) developed a PST model that includes the Learning-Modification-Application (LMA) approach, which itself includes seven stress distractions and biofeedback training (BFT). This process allows the modification and transformation of the psychological techniques included within routines to be applied in competition situations. This model has been used extensively in various sport disciplines and has demonstrated positive effects, mainly among elite athletes (Blumenstein and Orbach, 2018, 2023). By using PST that includes LMA, the transformation of imagery and relaxation in a self-paced task (i.e., free throws) is presented as a means to regulate emotional state in a basketball game.

The innovation of this PST model is its integration with the athlete’s/team’s training process and incorporation of the periodization principle, which is one of the most important concepts in training and planning (Bompa et al., 2019). Periodization consists of three major periods: preparatory (i.e., general and specific preparation, competition, and transition. Each involves different content and time lengths according to the demands of the sport and competition. Throughout the training program, psychological skills are learned and practiced, becoming more specific to the particular sport and the athlete’s/team’s practice.

The PST program is composed of five stages (see Table 1), which overlap with the LMA approach. In the first Introduction stage of the PST model, usually at the beginning of the sport season parallel to general preparation), the sport-psychology consultant visits the athlete’s practices during the first training month in the daily regime. The main goal for the consultant is to better understand the training process and the characteristics of the sport discipline through observation and conversations. Such cooperation with the sport-psychology consultant can allow the athlete/team and the coach to harmoniously cross to the next stage.

**TABLE 1:** Main intervention programs in the psychological skills training (PST) model.

	Preparatory phase			Competition phase	Transition phase
	General preparation		Specific preparation		
PST model	Introduction	Learning	Modification	Application	Analysis and Recovery
LMA stages		Learning	Modification	Application	
Stress distraction level		1–2	3–4	5–7	
Lab/field ratio%	20/80	70/30	50/50	30/70	60/40
Place of PST	Mainly in field	Lab	Lab and field	Field	Lab and competition results

The second stage of the PST model, the Learning stage, is provided parallel to general preparation during the Preparatory phase of an athlete’s training. The main objective of practice during general preparation is to strengthen the physical/technical/psychological foundations of the athlete. A high volume of training, long-duration practice sessions, and moderate intensity are the main characteristics of this aspect of training in most sports (Bompa et al., 2019). During the Learning stage the athlete practices the main psychological strategies/interventions, such as BFT, muscle relaxation, concentration, imagery, and self-talk. The process is usually accompanied by biofeedback (BF) control, such as heart rate (HR), electromyography (EMG), and electrodermal activity (EDA)/galvanic skin response (GSR). Moreover, basic psychological strategies are learned and practiced according to a stress distraction scale that was developed from applied work with BFT in a variety of athlete levels and sport disciplines (Blumenstein and Orbach, 2018).

During the Learning stage, the athlete masters and performs basic psychological strategies under “light” stress distractions (levels 1–2 on our 7-point scale) in laboratory settings.

*Stress distraction level 1*: Stress is produced by initial exposure to a BF device (e.g., electrodes, BF data, laboratory setting).

*Stress distraction level 2*: Stress is generated through verbal comments, such as positive and negative remarks during the athlete’s BF training.

The length of the Learning stage is approximately 2 months (7–8 sessions), during which time the sport-psychology consultant visits the athlete’s practice two or three times a week. For example, for relaxation skills, the athlete initially learns the basic version of relaxation and uses it for recovery purposes after intensive practice. At this stage, relaxation should last 10–15 mins. At the end of the stage the athlete should be able to use the relaxation skills independently during practice according to the demands of the sport discipline.

The third stage of the PST model is the Modification stage and is applied parallel to the specific preparation of Preparatory phase of the athlete’s training. The main objective of specific preparation is to further develop the athlete’s physical ability according to the unique physical and physiological characteristics of the sport (e.g., Blumenstein and Orbach, 2018; Bompa et al., 2019). Moreover, at this time the athlete integrates the technical and tactical components of their preparation into their practice. Consequently, the mental sessions and psychological strategies are modified according to the demands of practice. For example, during mental sessions, the athlete focuses on concentration and imagery techniques in which they visualize technical elements of themself or their opponents (e.g., in combat sports). Moreover, the length of the psychological interventions and their packaging are related to the sport discipline. The overall length of the Modification stage is approximately 2 months (7–8 sessions), which are provided in a lab/training setting under “moderate” stress distractions (level 3–4 on our 7-point scale). In these training settings, portable BF devices are used.

*Stress distraction level 3*: Stress is produced through specific demands, such as performance quality and time limits: (a) performing relaxation (or concentration) to achieve a specific value (e.g., difference from pre to post relaxation) in HR, EMG, or EDA/GSR measures; (b) the ability to relax or concentrate within a time limit of 0.5, 1, 3, or 5 mins. For example, the athlete may have two goals: to achieve relaxation with HR BF over 2 mins with a delta of 10 bpm (e.g., from 72 to 62 bpm) and to achieve frontalis (forehead) muscle EMG relaxation within the range of 2.4–1.4 μV during 1 min (Blumenstein et al., 2002).

*Stress distraction level 4*: Stress is produced under the same conditions as in the previous level, but with reward/punishment demands that are stressful to the athlete.

The fourth stage of the PST model is the Application stage, which is linked to the Competition phase of the periodization principle. The focus of this stage is to practice the technical/tactical elements of the athlete’s performance. The practice includes simulating previous competition events and generating real-life situations using a variety of stress distractions. Consequently, mental training sessions during this period include practice of skills such as short relaxation, fast concentration, and performance imagery with biofeedback control under competitive stress, and finally as part of PP-R. For this purpose, competitive noises and scenes are prepared and practiced (stress distractions level 5–7).

*Stress distraction level 5:* The stress at this level is generated by real-life competition sound clips. The athlete practices their mental skills under conditions that include spectator sounds, the referees’ remarks, competition music, and all other specific environmental and competition sounds.

*Stress distraction level 6:* Stress is produced through video clips of the athlete and their opponents. In addition, the video clips include winning/losing matches, races, successful/unsuccessful attempts, and starts of races.

*Stress distraction level 7****:*** Stress is produced through a combination of the stress distractions listed under levels 1–6.

The fifth and final stage of the PST model, Analysis and Recovery, includes analysis, recovery, and correction of possible mistakes. The objective of this stage is to help the athlete begin recovering from the extreme physical and psychological efforts they have made during the Competition phase. Included is an analysis of the positive and negative sides of competition results, together with relaxation techniques while listening to music. During this stage, it is recommended that a few individual/team sessions be conducted regarding future cooperation with the sport-psychology consultant and new goals for the next season.

Ultimately, practice of the 5-stage PST model helps the athlete “bring it all together” in critical moments of competition. It allows the athlete to integrate mental skills as part of PCA-R and PP-R for performance enhancement. The main aim of the following case study is to demonstrate how a preparatory routine is learned, practiced, and applied to a self-paced motor task, based on the 5-stage PST model.

## Case study: Pre-performance routine for shooting free-throw shots in basketball

Free-throw shots in basketball are performed during the game under conditions of fatigue and pressure. The stress level may fluctuate depending on the game’s situation. The player should quickly (i.e., 5–15 s) adjust their high arousal and physical effort to a lower level suitable to the shot and should find/recognize the balance between concentration and muscle relaxation. Studies have indicated a positive effect of PP-R, including physical routine, relaxation, imagery, and a combination of them, on the accuracy of free-throw shooting in basketball (Lidor, 2007; Lonsdale and Tam, 2008; Jackson, 2014; Blumenstein et al., 2016). In addition, the length of training time and order of the psychological techniques within PP-R for performance enhancement should be defined.

This case study is about an 18-year-old female (LO) who practiced in a professional basketball club and experienced a drop in her free-throw shooting percentage from 70 to 40%. The first author (IO) served at that time as the sport-psychology consultant for this club. The coach believed that the major obstacle LO faced was mental since the percentage drop occurred chiefly during critical game moments, and was concerned that LO did not implement a consistent and stable PP-R. Therefore, the coach approached IO and asked her to work with LO on improving her coping skills in stressful conditions. IO planned a mental training program for LO based on the 5-stage PST model, which she implemented after receiving the coach’s consent. Since IO had worked with this club for past last year, there was no need for the Introduction stage of the PST model.

During the Learning stage of the PST model, IO met with LO twice weekly in the lab and began teaching her basic psychological skills: relaxation, imagery, self-talk, concentration, and reframing based on attribution training principles. After about 3 weeks of practicing the basic mental skills, LO felt more comfortable, especially with progressive muscle relaxation exercises focusing on the upper body, imagery of relaxing scenes, and awareness of the connection between thoughts and feelings. Most of the training included BFT, using GSR/EDA (i.e., Learning stage of the LMA). LO reported that she practiced the mental skills approximately three times a week and as a result felt more comfortable with those she had acquired. During practice she attempted to apply the mental skills in relevant training situations, such as relaxation before the free throw and self-talk after mistakes.

During the third stage of the PST model, the Modification stage, which is associated with the Specific Preparation stage, the goal was to modify LO’s basic mental skills and psychological technique in order to develop a PP-R to use before the free-throw shots. The PP-R included fast muscle relaxation, quick concentration, verbal cues, imagery/simulation of the movement, and finally the shot itself. LO’s progress during this stage was tested using BF control with GSR (ultimate target delta of 450 within 30 s and 150 within 10 s under stressful demands), time reproduction exercises of 5 and 3 s under various stressors (e.g., moderate physical and mental stress; Weinberg and Gould, 2018), and imagery of free-throw shots (10 attempts per set). In addition, LO learned how to reframe her thoughts during (by using self-talk) and after (principles of attribution training) practice as a general mental skill for strong mental toughness. At the end of this stage, LO was expected to apply her PP-R during practice sessions. She usually stayed after the end of the practice, continuing to perform a combination of the PP-R and the shot itself.

During the fourth stage of the PST-model, the Application stage, which is associated with the Competition phase, it was possible to see not only improvement in LO’s free-throw shots, but also in her overall basketball performance. Her free-throw shots percentage increased to a consistent 60–65% under relatively higher competitive stress levels. In addition, she became a dominant player, receiving more than 25 min of playtime each game. The PP-R included quick relaxation, deep 1–2 breathing, dribbling, concentration (use of cue words), short imagery of the upcoming shot, and finally, the shot itself. After the first attempt at the shot, LO evaluated the success of the shot and, based on that, adjusted the PP-R accordingly for the second shot.

For the last stage of the PST-model, the Transition stage, three different situations required three different applications. The first situation was transition after a game followed by a 1-day rest. During this time, LO rested and used the day off to do things that would conserve her physical and mental state: practicing relaxation for the body and the mind. The second situation was transition half-way through the league schedule, when LO rested for 7 days. During this time, she met with IO a few times while practicing her shots and participated in enjoyable events. Finally, transition at the end of the season lasted for 3 weeks. At this time LO’s goal was to “enjoy” her successful season, analyze and remember the actions she took to achieve her goals, and most importantly, practice long physical relaxation sessions together with general fitness exercises.

## Discussion and summary

Preparatory routine comprises behavioral and mental components through which athletes attempt to regulate their emotions and actions (Jackson, 2014). To achieve this goal, all mental techniques within PR are first learned in a laboratory and then modified and transformed according to competitive demands and the sport discipline. Finally, the motor process can be applied automatically with minimal conscious interferences. The 5-stage PST model can be used as a conceptual framework for learning, practicing, and applying PRs in an optimal and efficient manner (Blumenstein and Orbach, 2018, 2023). The availability of biofeedback measurements in the model provides athletes with relevant information regarding their emotional regulation. Therefore, using biofeedback training can be helpful for transferring learned psychological skills from laboratory to field. The case study presented in this article is an example of incorporating mental-skills training based on the 5-stage PST model into physical/training processes. It demonstrates how PR can be used for emotional regulation in performance enhancement, especially in self-paced motor tasks such as basketball free throws.

The benefits of this model are critical for athletes who can use it to learn, practice, and perform their tasks in a consistent matter. In addition, it is important for coaches, so they can be aware of an essential tool for emotional regulation in performance enhancement. Future studies should continue to investigate the usefulness of the 5-stage PST model for different ages, genders, and skill levels.

## Data availability statement

The raw data supporting the conclusions of this article will be made available by the authors, without undue reservation.

## Author contributions

The ideas presented in the manuscript are based on a mutual work of IO and BB for the last 13 years. IO and BB contributed to the development of the manuscript idea, wrote together the initial draft of the perspective part of the manuscript while IO wrote the first draft of the case study and contributed to manuscript revisions. All authors contributed to the article and approved the submitted version.
